# Blockade of glycolysis-dependent contraction by oroxylin a via inhibition of lactate dehydrogenase-a in hepatic stellate cells

**DOI:** 10.1186/s12964-019-0324-8

**Published:** 2019-02-11

**Authors:** Feixia Wang, Yan Jia, Mengmeng Li, Ling Wang, Jiangjuan Shao, Qinglong Guo, Shanzhong Tan, Hai Ding, Anping Chen, Feng Zhang, Shizhong Zheng

**Affiliations:** 1 Jiangsu Key Laboratory for Pharmacology and Safety Evaluation of Chinese Materia Medica, School of Pharmacy, Nanjing University of Chinese Medicine, Nanjing, 210023 China; 2 Jiangsu Key Laboratory of Therapeutic Material of Chinese Medicine, School of Pharmacy, Nanjing University of Chinese Medicine, Nanjing, 210023 China; 3 State Key Laboratory Cultivation Base for TCM Quality and Efficacy, School of Pharmacy, Nanjing University of Chinese Medicine, Nanjing, 210023 China; 4 Jiangsu Key Laboratory of Carcinogenesis and Intervention, China Pharmaceutical University, Nanjing, 210009 China; 5 The Nanjing Hospital Affiliated to Nanjing University of Chinese Medicine, Nanjing, 210003 China; 6 Department of Pathology, School of Medicine, Saint Louis University, Saint Louis, MO 63104 USA

**Keywords:** Liver fibrosis, Oroxylin a, Hepatic stellate cell, Aerobic glycolysis, Contraction, Lactate dehydrogenase-a

## Abstract

**Background:**

Contraction of hepatic stellate cells (HSCs) plays an important role in the pathogenesis of liver fibrosis by regulating sinusoidal blood flow and extracellular matrix remodeling. Here, we investigated how HSC contraction was affected by the natural compound oroxylin A, and elucidated the underlying mechanism.

**Methods:**

Cell contraction and glycolysis were examined in cultured human HSCs and mouse liver fibrosis model upon oroxylin A intervention using diversified cellular and molecular assays, as well as genetic approaches.

**Results:**

Oroxylin A limited HSC contraction associated with inhibiting myosin light chain 2 phosphorylation. Oroxylin A blocked aerobic glycolysis in HSCs evidenced by reduction in glucose uptake and consumption and lactate production. Oroxylin A also decreased extracellular acidification rate and inhibited the expression and activity of glycolysis rate-limiting enzymes (hexose kinase 2, phosphofructokinase 1 and pyruvate kinas type M2) in HSCs. Then, we identified that oroxylin A blockade of aerobic glycolysis contributed to inhibition of HSC contraction. Furthermore, oroxylin A inhibited the expression and activity of lactate dehydrogenase-A (LDH-A) in HSCs, which was required for oroxylin A blockade of glycolysis and suppression of contraction. Oral administration of oroxylin A at 40 mg/kg reduced liver injury and fibrosis, and inhibited HSC glycolysis and contraction in mice with carbon tetrachloride-induced hepatic fibrosis. However, adenovirus-mediated overexpression of LDH-A significantly counteracted the oroxylin A’s effects in fibrotic mice.

**Conclusions:**

Blockade of aerobic glycolysis by oroxylin A via inhibition of LDH-A reduced HSC contraction and attenuated liver fibrosis, suggesting LDH-A as a promising target for intervention of hepatic fibrosis.

**Electronic supplementary material:**

The online version of this article (10.1186/s12964-019-0324-8) contains supplementary material, which is available to authorized users.

## Background

Hepatic fibrosis is a compensatory repair process in response to a variety of chronic liver injuries. Current paradigm has established that hepatic stellate cells (HSCs) are key effector cells in the initiation and development of hepatic fibrosis [1]. In fibrogenic liver, the quiescent HSCs undergo transdifferentiation into myofibroblasts with high proliferative and migratory capacities, and subsequently secrete massive extracellular matrix molecules, accumulating in liver parenchyma and promoting the pathogenesis of hepatic fibrosis [2]. Recent recognition of HSCs as liver-specific pericytes with contractile property is a key milestone in understanding of the biology of these cells [3]. HSCs regulate sinusoidal resistance and blood flow around sinusoids by contraction [4]. In addition, the contractile force generated by HSCs aggravates extracellular matrix remodeling during chronic liver injury [5]. Therefore, elucidating how HSC contraction is regulated may facilitate the development of therapeutic strategies for chronic liver disease.

Cell contraction involves dynamic synthesis and decomposition of actin and formation of large cytoskeletal structures [6]. When cells contract, the myosin cross-bridge periodically binds to actin, which dissociates and hydrolyzes ATP, releasing energy for actin filaments [7]. Cell contraction is thus a highly energy-consuming process. It has been well established that a key metabolic hallmark of cancer cells is aerobic glycolysis, termed Warburg effect [8]. Although glycolysis produces less ATP than oxidative phosphorylation does, the Warburg effect favors cell growth by rapidly providing ATP and carbon sources [8]. Glycolysis has some rate-limiting enzymes including hexokinase 2 (HK2), phosphofructokinase 1 (PFK1) and pyruvate kinas type M2 (PKM2) successively, converting glucose to pyruvate [9]. Notably, final conversion of pyruvate to lactate is a crucial step catalyzed by lactate dehydrogenase (LDH), of which LDH-A is a major subtype [10]. High expression or activity of LDH-A allows for rapid glycolysis flux so as to meet the energy demands of rapidly proliferating cells [11]. Recent evidence suggests that the activated HSCs are similar to the highly proliferative cancer cells with regard to their biosynthetic and bioenergetic requirements [12]. Aerobic glycolysis is a striking metabolic phenotype of activated HSCs during liver fibrosis [12]. However, little is known about the role of aerobic glycolysis in controlling of HSC contraction.

Natural products have been an importance source of drug candidates nowadays. Oroxylin A is an attractive natural compound with promising pharmacological activities. For example, oroxylin A was found to inhibit the growth and proliferation of hepatoma cells [13, 14]. Oroxylin A could also reduce glucose uptake and lactate production in HepG2 cells under hypoxia [15], and inhibit glycolysis-dependent growth of human breast tumors [16]. Our previous studies demonstrated that oroxylin A reduced liver fibrosis associated with induction of HSC autophagy [17]. We hold that the mechanisms underlying the antifibrotic effects of oroxylin A have not been fully understood. Here, we investigated whether and how oroxylin A affected HSC contraction with a focus on the association of aerobic glycolysis.

## Methods

### Chemicals and antibodies

Oroxylin A (HPLC purity 99.9%) was kindly provided by Professor Qinglong Guo (China Pharmaceutical University, Nanjing, China). Compounds 2-deoxy-D-glucose (2-DG) and galloflavin were purchased from Apexbio Technology (Houston, TX, USA). These reagents were dissolved with dimethylsulfoxide at indicated concentrations for in vitro experiments. The following primary antibodies were used for Western blot analysis in current study: antibodies against HK2, PFK1, PKM2, LDH-A, β-actin and GAPDH were obtained from Proteintech Group (Chicago, IL, USA); antibodies against p-MLC2^Ser19^, MLC2, α-SMA, fibronectin and α1(I) procollagen were obtained from Cell Signaling Technology (Danvers, MA, USA). Horseradish peroxidase-conjugated anti-mouse and anti-rabbit secondary antibodies were obtained from Proteintech Group (Chicago, IL, USA).

### Cell culture and transfection

Human HSC line LX2 cells and human normal hepatocyte line LO2 cells were obtained from the Cell Bank of Chinese Academy of Sciences (Shanghai, China). Cells were characterized using human short tandem repeat markers. Cells were cultured in Dulbecco’s modified eagle medium (Invitrogen, Grand Island, NY, USA) with 10% fetal bovine serum (Wisent Biotechnology Co., Ltd., Nanjing, China), 1% antibiotics, and grown in a 5% CO_2_ humidified atmosphere at 37 °C. LDH-A siRNA (sc-43,893) and control siRNA (sc-37,007) were obtained from Santa Cruz Biotechnology (Santa Cruz, CA, USA). LDH-A overexpression plasmid pcDNA3.1(+)-LDH-A was constructed by Jiangsu KeyGEN Biotechnology Co. Ltd. (Nanjing, China). Transfection with LDH-A siRNA or overexpression plasmid was performed using the Lipofectamine 2000 Transfection Reagent (Life Technologies, Grand Island, NY, USA) according to the protocols provided by the manufacturer.

### Collagen gel contraction assay

Collagen gel contraction assays were performed as we previously described [18]. Percentages of original gel area were quantified using the Image J software (Media Cybernetics, Rockville, MD, USA). Representative views are shown.

### Cytoskeleton staining

Cytoskeleton was visualized using FITC-conjugated phalloidin (Beyotime Biotechnology, Haimen, China) according to our previously described methods [18]. The nuclei of cells were stained with diamidino-phenyl-indole (DAPI). Photographs were blindly taken at five random fields under a microscope (ZEISS Axio vert. A1, Germany). Representative views are shown.

### Immunofluorescence staining

Staining with LX2 cells or mouse liver tissues was performed according to our descried methods [19]. The nuclei of cells were stained with DAPI. Photographs were blindly taken at five random fields under a microscope (ZEISS Axio vert. A1, Germany). Representative views are shown.

### Glucose uptake assay

The glucose uptake by LX2 cells was determined using a Glucose Uptake Assay Kit (Abnova, Taiwan, China) according to the manufacturer’s instructions. In this assay, the glucose analog 2-DG is metabolized to 2-DG-6-phosphate, which is proportional to glucose uptake by cells. The accumulated 2-DG-6-phosphate is enzymatically coupled to generate NADPH, which is specifically monitored by a NADPH sensor. The signal can be read by an absorbance microplate reader by reading the OD ratio at wavelength 570 to 610 nm.

### Glucose consumption assay

The glucose consumption by LX2 cells was determined using an enzyme-linked immunosorbent assay kit (Shanghai Meilian Biology Technology Co. Ltd., Shanghai, China) for measuring glucose oxidase (GOD) activity according to the protocols provided by the manufacture. GOD is an endogenous oxido-reductase efficiently catalyzing the oxidization of glucose into gluconic acid. Its activity is an alternative indicator of glucose consumption [20].

### Measurement of lactate levels

Lactate levels in lysates of LX2 cells or mouse liver tissues were measured using kits (Nanjing Jiancheng Bioengineering Institute, Nanjing, China) following the manufacturer’s instructions.

### Measurement of extracellular acidification rate (ECAR)

ECAR was measured using a pH-Xtra™ Glycolysis Assay Kit from Luxcel Biosciences (Cork, Ireland) following the manufacturer’s instructions and reported methods [21]. The pH-Xtra™ assay uses a pH-sensitive fluorophore which detects acidification due to glycolysis-related release of lactate.

### Measurement of intracellular ATP levels

Intracellular ATP levels were determined using an ATP Assay Kit provided by Beyotime Institute of Biotechnology (Haimen, China) according to the protocols provided by the manufacture.

### Enzyme activity assay

The intracellular activities of HK2, PFK1, PKM2 and LDH-A in LX2 cell were measured using kits (Shanghai Meilian Biology Technology Co. Ltd., Shanghai, China) according to the protocols provided by the manufacture.

### Cell viability assay

The viability of LX2 cells or LO2 cells treated with 2-DG or galloflavin was evaluated using MTT assays. Briefly, the medium of treated cells was replaced with 100 μl phosphate buffered saline containing 0.5 mg/ml MTT and then was incubated at 37 °C for 4 h. The crystals were dissolved with 200 μl dimethylsulfoxide. The spectrophotometric absorbance at 490 nm was measured by a SPECTRAmax™ microplate spectrophotometer (Molecular Devices, Sunnyvale, CA, USA). Cell viability was expressed as percentage of control.

### Human liver samples

Liver samples from five healthy subjects and five patients with liver fibrosis were provided by the Nanjing Hospital Affiliated to Nanjing University of Chinese Medicine (Nanjing, China). The study followed the tenets of the Declaration of Helsinki, and informed written consents were obtained from all patients followed by explanation of the nature and possible consequences of the study. The study protocol was approved by the Medical Ethical Committee of the Second Hospital of Nanjing.

### Animal procedures and treatments

Animal experimental procedures were approved by the Institutional and Local Committee on the Care and Use of Animals of Nanjing University of Chinese Medicine, and all animals were received humane care according to the National Institutes of Health (USA) guidelines. Thirty male ICR mice (8-week old) were obtained from Shanghai Slac Laboratory Animal Co., Ltd. (Shanghai, China). Mice were housed in standardized conditions at 20 ± 2 °C room temperature, 40 ± 5% relative humidity and a 12 h light/dark cycle. A mixture of carbon tetrachloride (CCl_4_) and olive oil [2:3 (*v*/v)] was used to induce hepatic fibrosis in mice via intraperitoneal injection (0.1 ml/100 g body weight). Thirty mice were randomly divided into five groups (*n* = 6): (1) control, (2) model, (3) oroxylin A treatment, (4) oroxylin A treatment plus adenovirus vector, and (5) oroxylin A treatment plus LDH-A plasmid adenovirus (constructed by OBiO Technology Co. Ltd., Shanghai, China). Initially, mice in groups 4 and 5 were received caudal vein injection with corresponding adenovirus once. Two weeks later, mice in groups 2–5 were received intraperitoneal injection with CCl_4_ every three days for 4 weeks. Simultaneously, mice in groups 3–5 were orally given oroxylin A suspended in CMC-Na solution at 40 mg/kg once daily for 4 weeks. This dose was determined by preliminary experiments. Mice in group 1 were orally given equal amount of CMC-Na solution once daily and injected with olive oil intraperitoneally every three days for 4 weeks, and mice in group 2 were also orally given equal amount of CMC-Na solution once daily for 4 weeks. At the end of experiments, all mice were anesthetized with isoflurane followed by blood collection via retro orbital sinus and isolation of liver.

### Serum biochemistry

Serum levels of alanine aminotransferase (ALT), aspartate aminotransferase (AST), total bilirubin (TBIL), indirect bilirubin (IBIL), hyaluronic acid (HA), laminin (LN) and procollagen type III (PC-III) were measured using kits (Nanjing Jinting Bioengineering Institute, Nanjing, China) according to the protocols provided by the manufacture.

### Measurement of hepatic hydroxyproline (Hyp)

The Hyp levels in mouse liver tissues were measured using a kit (Nanjing Jiancheng Bioengineering Institute, Nanjing, China) according to the protocols provided by the manufacture.

### Liver histopathology and collagen staining

Mouse liver tissues were fixed in 10% neutral buffered formalin and embedded in paraffin. Hematoxylin-eosin (H&E) staining was used for assessment of histopathology according to standard methods. Masson staining and Sirius Red staining were used for exanimation of collagens according to standard methods. Photographs were blindly taken at five random fields under a microscope (ZEISS Axio vert. A1, Germany). Representative views are shown.

### Immunohistochemistry

Mouse liver tissue sections were incubated with primary antibody against α-smooth muscle actin (α-SMA) for immunohistochemical evaluation using standard methods. Photographs were blindly taken at five random fields under a microscope (ZEISS Axio vert. A1, Germany). Representative views are shown.

### Scanning electronic microscopy (SEM)

Sinusoidal fenestration of mouse liver was examined by SEM according to our previously reported methods [19]. Photographs were blindly taken at five random fields, and representative images are shown.

### Real-time PCR

Total RNA was extracted from LX2 cells, mouse liver tissues, or human liver samples using Trizol reagent (Sigma, Saint Louis, MO, USA). Total RNA was subject to reverse transcription to cDNA using the TransScript All-in One First-Strand cDNA Synthesis SuperMix for qPCR (One-Step gDNA Removal) Kits provided by TransGen Biotech Co., Ltd. (Beijing, China) according to the protocols. Real-time PCR was performed using the SYBR Green Master Mix (Vazyme Biotech Co., Ltd., Nanjing China) according to the protocol. Fold changes in the mRNA levels of target genes were related to the invariant control glyceraldehyde phosphate dehydrogenase (GAPDH). The primers (GenScript Co., Ltd., Nanjing, China) are listed in Additional file 1: Table S1.

### Western blot assay

Whole cell protein extracts were prepared from LX2 cells or mouse liver tissues with RIPA buffer containing protease inhibitors and phosphatase inhibitors. Protein detection and band visualization and quantification were performed as we previously described [22]. β-Actin or GAPDH was used as an invariant control for equal loading of total proteins. Representative blots were shown.

### Statistical analysis

Data from at least triplicate experiments are presented as mean ± SD. One-way ANOVA was performed to analyze the data using GraphPad Prism 7 (San Diego, CA, USA). In all cases, a *P* value of 0.05 or lower was considered significant.

## Results

### Oroxylin a inhibits HSC contraction

**Fig. 1:**
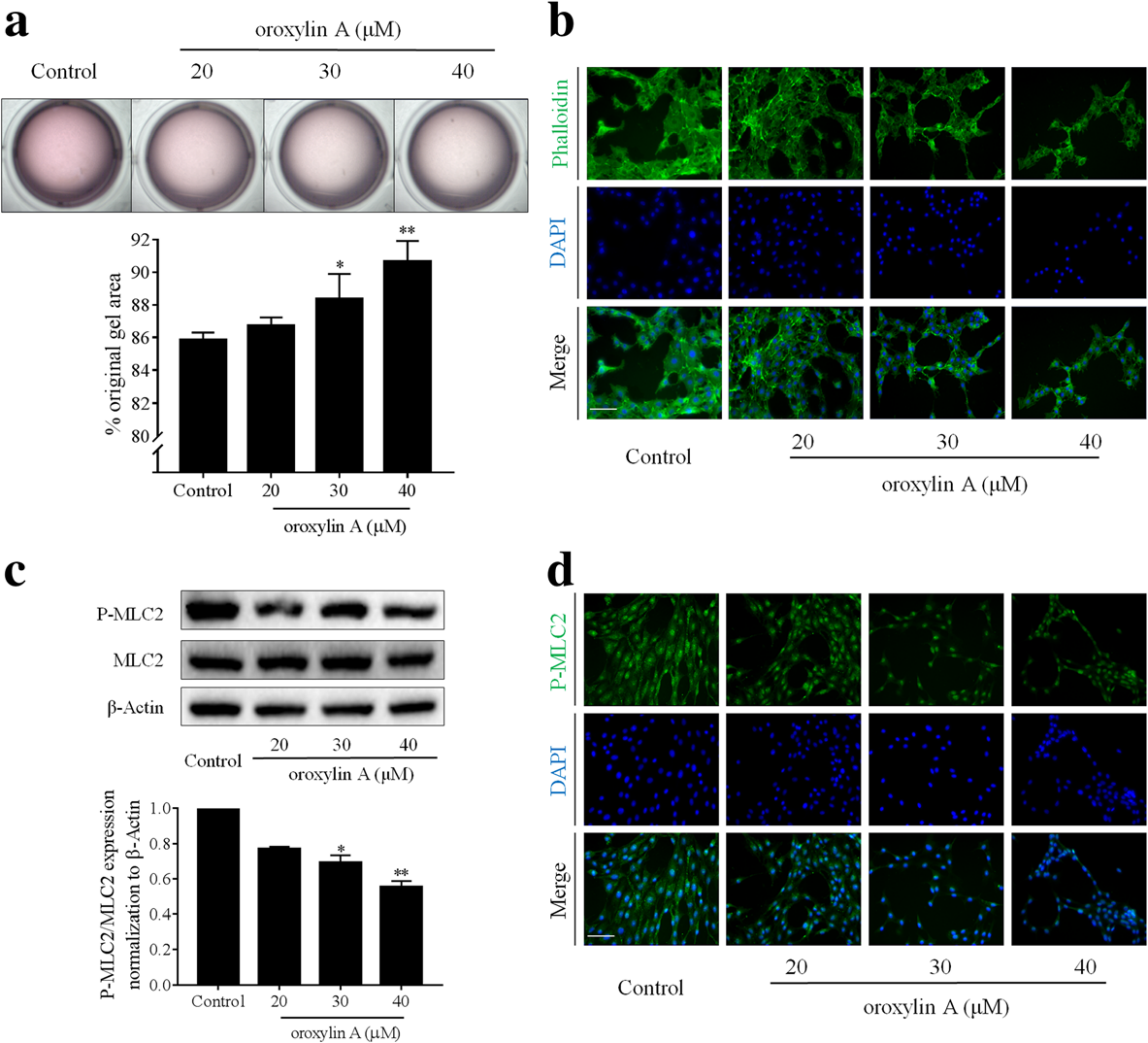
Oroxylin A inhibits HSC contraction. LX2 cells were treated with oroxylin A at indicated concentrations for 24 h. **a** Collagen gel contraction assays with quantification. **b** Cytoskeleton fluorescence staining, scale bar: 20 μm. **c** Western blot analyses of MLC2 phosphorylation with quantification. **d** Immunofluorescence analyses of MLC2 phosphorylation, scale bar: 20 μm. For statistical significance of this figure: *p < 0.05 vs. control, **p < 0.01 vs. control

Results of collagen gel contraction assays showed that oroxylin A concentration-dependently inhibited HSC contraction, and that oroxylin A at 30 and 40 μM produced significant effects (Fig. 1a). The organization of cell contractile machinery can be manifested by cytoskeleton arrangement [23]. Cytoskeleton fluorescence staining revealed that oroxylin A reduced the formation of actin stress fibers and disturbed the microfilament skeleton in a concentration-dependent manner in HSCs (Fig. 1b). Phosphorylation of myosin light chain 2 (MLC2) is an important event during cell contraction [24]. Western blot analyses demonstrated that oroxylin A concentration-dependently decreased MLC2 phosphorylation in HSCs (Fig. 1c). Immunofluorescence analyses of MLC2 phosphorylation provided consistent results (Fig. 1d). Together, these data indicated that oroxylin A inhibited HSC contraction.

### Oroxylin a blocks aerobic glycolysis leading to inhibition of HSC contraction

**Fig. 2:**
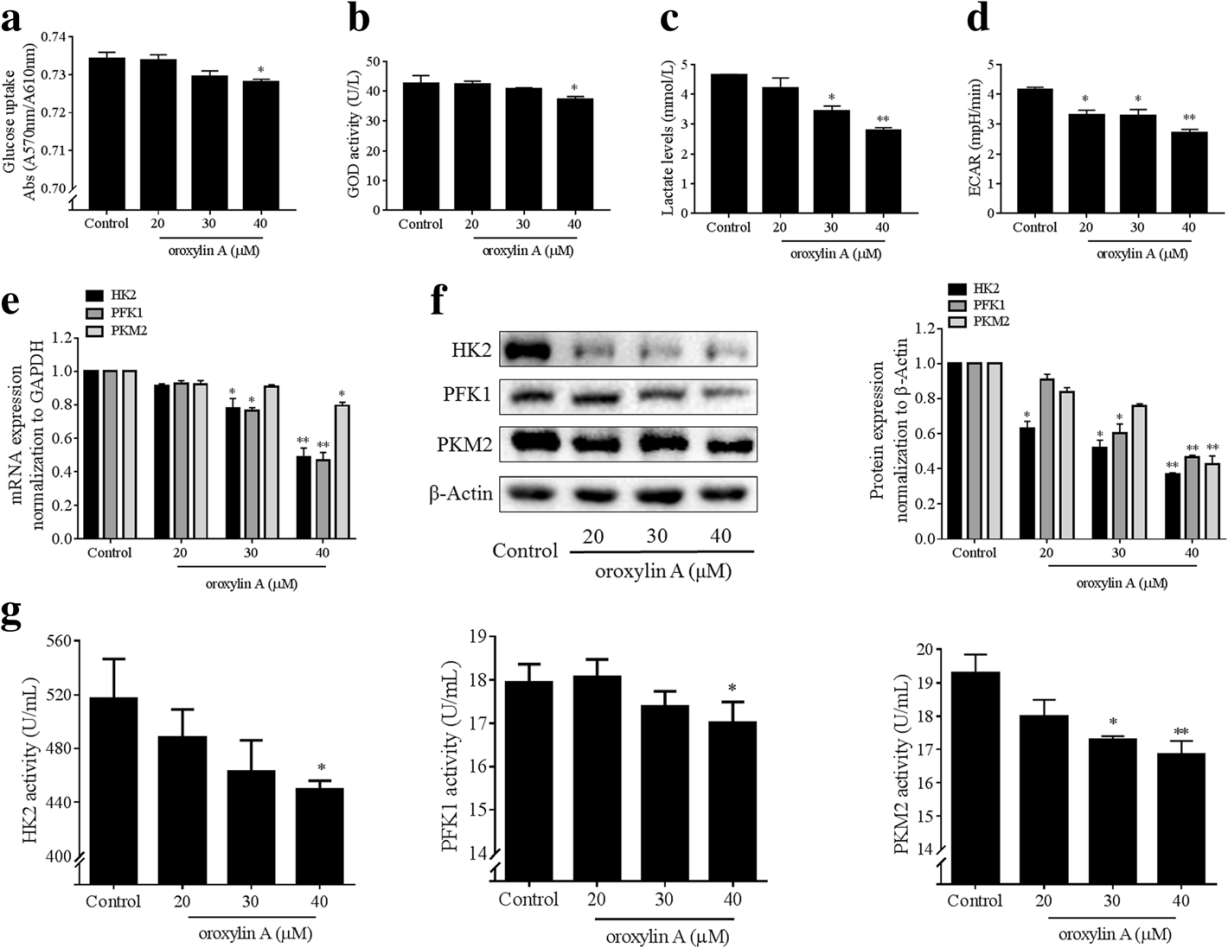
Oroxylin A blocks aerobic glycolysis in HSCs. LX2 cells were treated with oroxylin A at indicated concentrations for 24 h. **a** Measurements of glucose uptake. **b** Measurements of glucose consumption indicated by GOD activity. **c** Measurements of intracellular lactate levels. **d** Measurements of ECAR. **e** Real-time PCR analyses of mRNA expression of HK2, PFK1 and PKM2. **f** Western blot analyses of protein expression of HK2, PFK1 and PKM2 with quantification. **g** Measurements of intracellular enzyme activities of HK2, PFK1 and PKM2. For statistical significance of this figure: *p < 0.05 vs. control, **p < 0.01 vs. control

We then tested the effects of oroxylin A on aerobic glycolysis of HSCs. We observed that oroxylin A decreased glucose uptake (Fig. 2a) and glucose consumption indicated by GOD activity (Fig. 2b) in a concentration-dependent fashion in HSCs. Lactate production and ECAR were also reduced by oroxylin A concentration-dependently (Fig. 2c, d). We further detected the effects of oroxylin A on three rate-limiting enzymes HK2, PFK1 and PKM2, and observed that the mRNA and protein expression of these enzymes were downregulated by oroxylin A in HSCs (Fig. 2e, f). Meanwhile, oroxylin A decreased the intracellular activities of HK2, PFK1 and PKM2 (Fig. 2g). Additional data showed that the intracellular ATP levels were reduced by oroxylin A concentration-dependently in HSCs (Additional file 2: Figure S1). The above findings collectively revealed that the overall glycolytic flux and activity were effectively blocked by oroxylin A, cutting of the energy supply within HSCs.

**Fig. 3:**
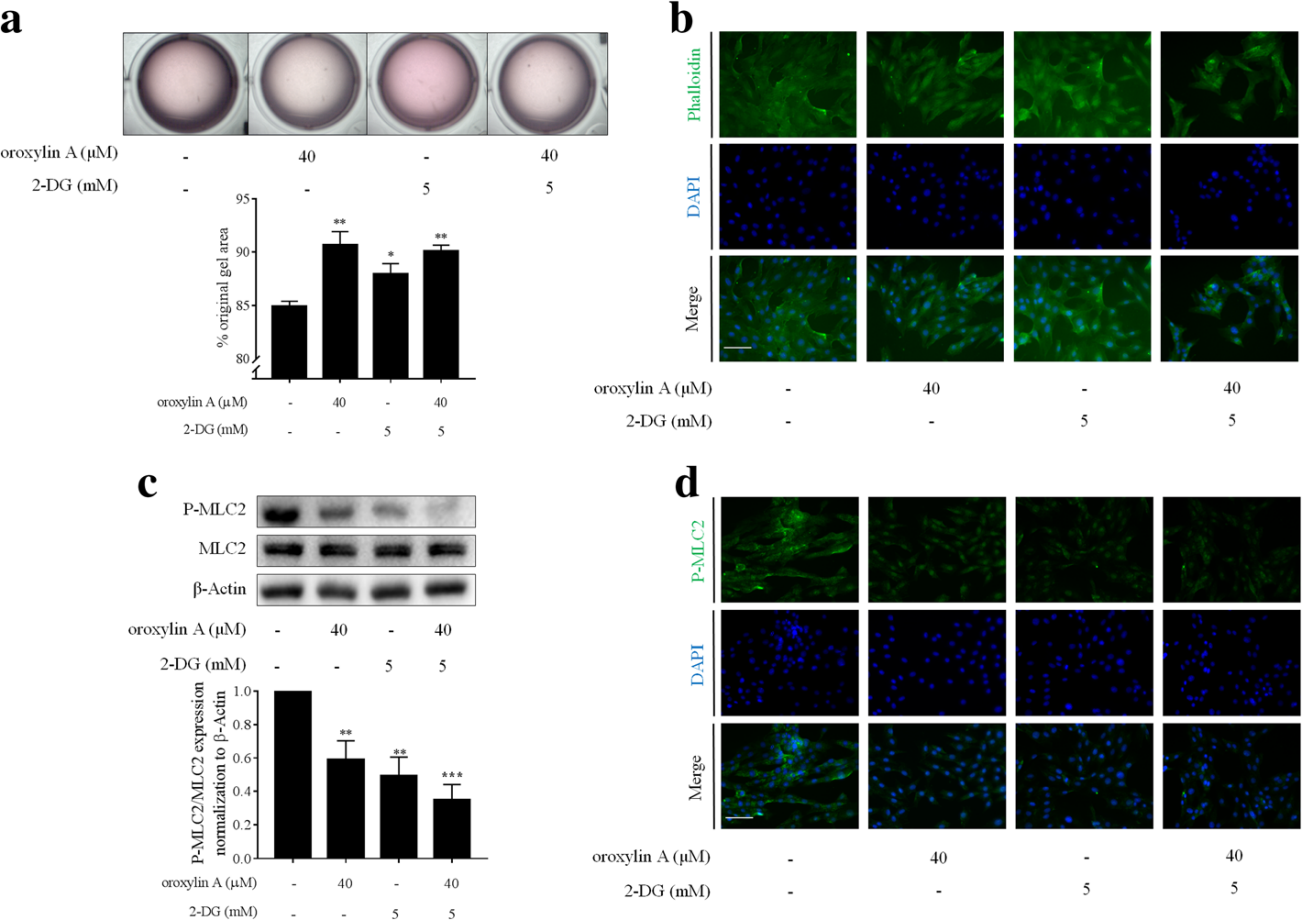
Oroxylin A blockade of aerobic glycolysis led to inhibition of HSC contraction. LX2 cells were treated with oroxylin A and/or 2-DG at indicated concentrations for 24 h. **a** Collagen gel contraction assays with quantification. **b** Cytoskeleton fluorescence staining, scale bar: 20 μm. **c** Western blot analyses of MLC2 phosphorylation with quantification. **d** Immunofluorescence analyses of MLC2 phosphorylation, scale bar: 20 μm. For statistical significance of this figure: *p < 0.05 vs. control, **p < 0.01 vs. control, ***p < 0.001 vs. control

We next asked whether blockade of aerobic glycolysis was associated with the reduced contractile capacity in oroxylin A-treated HSCs. The glycolysis inhibitor 2-DG was used to test the association. We used 2-DG at 5 mM for experiments based on the observation that 2-DG at this concentration suppressed HSC viability but did not affect hepatocyte viability (Additional file 3: Figure S2a, b). Collagen gel contraction assays showed that 2-DG at 5 mM, similar to oroxylin A at 40 μM, significantly suppressed HSC contraction, and their combination produced more potent inhibitory effects (Fig. 3a). Cytoskeleton fluorescence staining revealed that microfilament skeleton was disrupted by 2-DG and its combination with oroxylin A (Fig. 3b). Examinations of MLC2 phosphorylation using Western blot analysis and immunofluorescence staining consistently exhibited that 2-DG at 5 mM alone, or combined with oroxylin A at 40 μM, significantly downregulated the phosphorylation levels of MLC2 in HSCs (Fig. 3c, d). Altogether, these results indicated that blockade of aerobic glycolysis by oroxylin A resulted in the suppression of HSC contraction.

### Oroxylin a inhibits LDH-A in HSCs

**Fig. 4:**
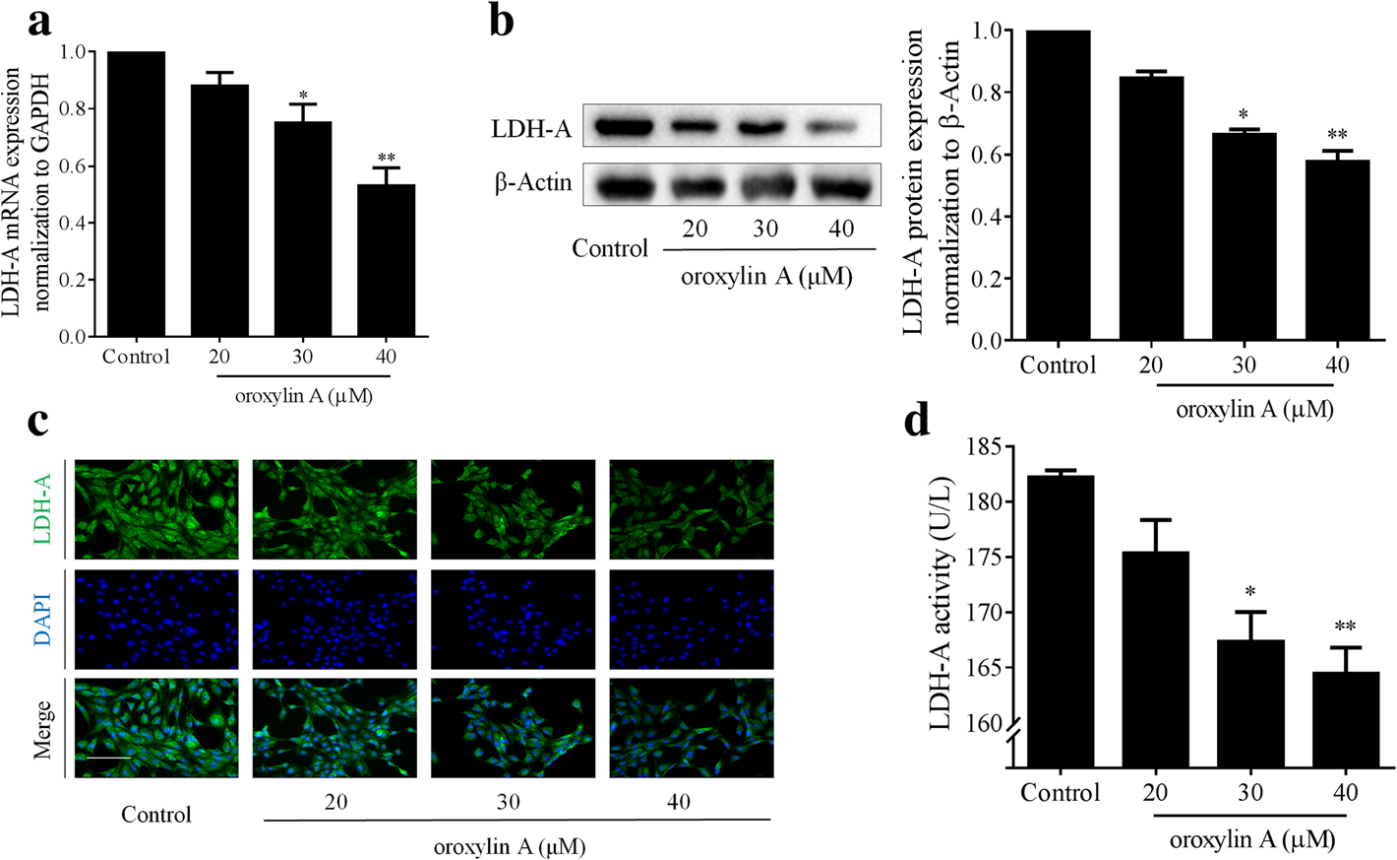
Oroxylin A inhibits LDH-A in HSCs. LX2 cells were treated with oroxylin A at indicated concentrations for 24 h. **a** Real-time PCR analyses of mRNA expression of LDH-A. **b** Western blot analyses of protein expression of LDH-A with quantification. **c** Immunofluorescence analyses of LDH-A expression. **d** Measurements of intracellular LDH-A activity. For statistical significance of this figure: *p < 0.05 vs. control, **p < 0.01 vs. control

Given that LDH-A is a central player in glycolysis and has a multifunctional role in cell biology [25], we next focused on the regulation of LDH-A by oroxylin A in HSCs. We observed that the mRNA levels of LDH-A in liver tissues from patients with hepatic fibrosis were significantly higher than that in healthy liver tissues (Additional file 4: Figure S3), strongly suggesting the role of LDH-A in the pathology of liver fibrosis. We then found that the LDH-A mRNA expression was reduced by oroxylin A in a concentration-dependent manner in cultured HSCs (Fig. 4a). Oroxylin A also downregulated the protein abundance of LDH-A in HSCs evidenced by Western blot and immunofluorescence analyses (Fig. 4b, c). Consistently, the intracellular enzyme activity of LDH-A was decreased by oroxylin A concentration-dependently (Fig. 4d). Taken together, these results revealed that oroxylin A inhibited the expression and activity of LDH-A in HSCs.

### Inhibition of LDH-A is required for oroxylin a to block aerobic glycolysis and reduce contraction in HSCs

**Fig. 5:**
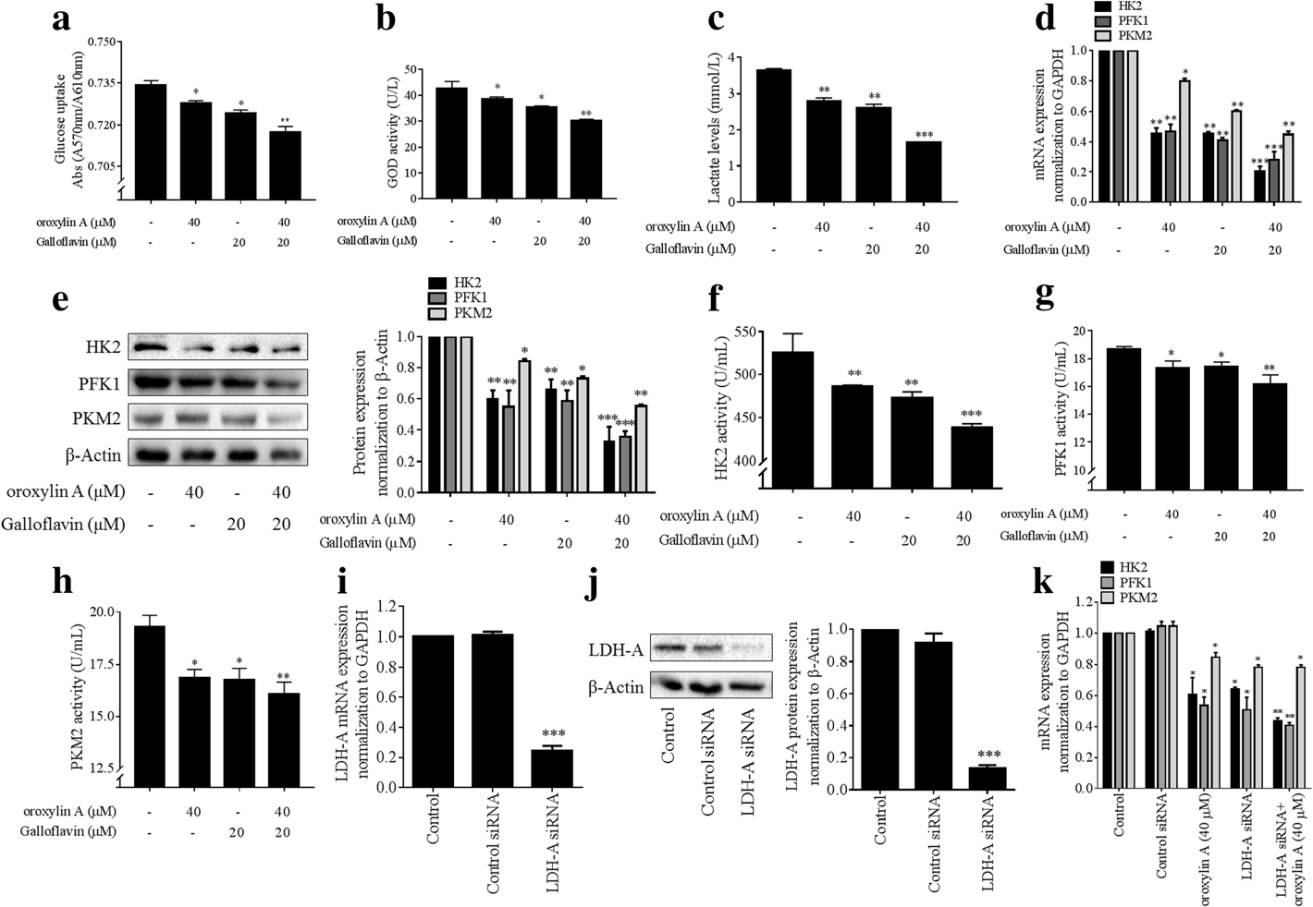
Oroxylin A inhibition of LDH-A led to blockade of aerobic glycolysis in HSCs. LX2 cells were treated with oroxylin A and/or galloflavin at indicated concentrations, or transfected with LDH-A siRNA for 24 h. **a** Measurements of glucose uptake. **b** Measurements of glucose consumption indicated by GOD activity. **c** Measurements of intracellular lactate levels. **d** Real-time PCR analyses of mRNA expression of HK2, PFK1 and PKM2. **e** Western blot analyses of protein expression of HK2, PFK1 and PKM2 with quantification. **f** Measurements of intracellular HK2 enzyme activity. **g** Measurements of intracellular PFK1 enzyme activity. **h** Measurements of intracellular PKM2 enzyme activity. **i** Real-time PCR analyses of mRNA expression of LDH-A. **j** Western blot analyses of protein expression of LDH-A with quantification. **k** Real-time PCR analyses of mRNA expression of HK2, PFK1 and PKM2. For statistical significance of panels a-h: *p < 0.05 vs. control, **p < 0.01 vs. control, ***p < 0.001 vs. control. For statistical significance of panels i-k: *p < 0.05 vs. control siRNA, **p < 0.01 vs. control siRNA, ***p < 0.001 vs. control siRNA

The above results suggested LDH-A as a potential target molecule for oroxylin A in HSCs, and we subsequently attempted to confirm this hypothesis. The compound galloflavin, a selective pharmacological inhibitor of LDH-A [26], was used to test the role of LDH-A in oroxylin A blockade of aerobic glycolysis. We used galloflavin at 20 μM for experiments based on the observation that galloflavin at this concentration inhibited HSC viability but did not affect hepatocyte viability (Additional file 3: Figure S2c, d). We observed that galloflavin at 20 μM, similar to oroxylin A at 40 μM, significantly inhibited glucose uptake and consumption and reduced the production of lactate in HSCs, and that combination of galloflavin and oroxylin A produced more potent reducing effects on these parameters (Fig. 5a-c). Further examinations of glycolysis rate-limiting enzymes showed that galloflavin at 20 μM significantly decreased the expression and activities of HK2, PFK1 and PKM2 in HSCs, and that its combination with oroxylin A resulted in more potent inhibitory effects on these enzymes (Fig. 5d-h). To confirm the results, HSCs were transfected with LDH-A siRNA to knockdown LDH-A expression at both mRNA and protein levels (Fig. 5i, j). Consistently, transfection with LDH-A siRNA alone or combined with oroxylin A treatment significantly downregulated the mRNA expression of HK2, PFK1 and PKM2 (Fig. 5k). Additionally, overexpression of LDH-A increased the expression of HK2, PFk1 and PKM2 and rescued oroxylin A-induced reduction of these enzymes in HSCs (Additional file 5: Figure S4). Overall, genetic deficiency of LDH-A or pharmacological inhibition of LDH-A effectively diminished the glycolytic activity in HSCs, and synergistic effects could be achieved when combined with oroxylin A, suggesting that inhibition of LDH-A was required for oroxylin A to block aerobic glycolysis.

**Fig. 6:**
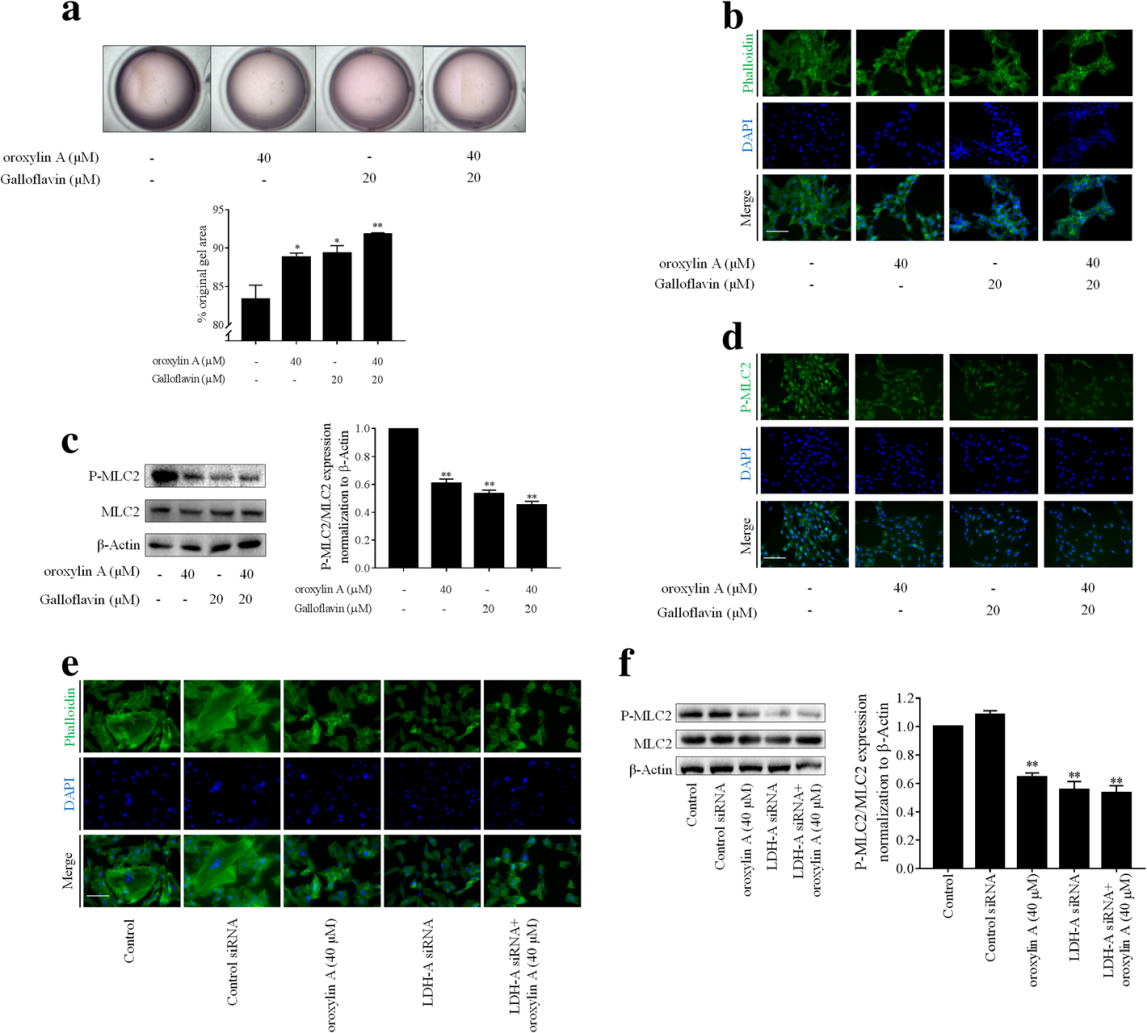
Oroxylin A inhibition of LDH-A led to reduction of HSC contraction. LX2 cells were treated with oroxylin A and/or galloflavin at indicated concentrations, or transfected with LDH-A siRNA for 24 h. **a** Collagen gel contraction assays with quantification. **b** Cytoskeleton fluorescence staining, scale bar: 20 μm. **c** Western blot analyses of MLC2 phosphorylation with quantification. **d** Immunofluorescence analyses of MLC2 phosphorylation, scale bar: 20 μm. **e** Cytoskeleton fluorescence staining, scale bar: 20 μm. **f** Western blot analyses of MLC2 phosphorylation with quantification. For statistical significance of panels a-d: *p < 0.05 vs. control, **p < 0.01 vs. control. For statistical significance of panels e-f: **p < 0.01 vs. control siRNA

We further investigated the association between inhibition of LDH-A and suppression of contraction by oroxylin A in HSCs. As expected, collagen gel contraction assays and cytoskeleton fluorescence staining showed that galloflavin at 20 μM, similar to oroxylin A at 40 μM, significantly reduced HSC contraction, and that combination of the two compounds produced more potent effects (Fig. 6a, b), which were confirmed by analyses of MLC2 phosphorylation by Western blot and immunofluorescence assays (Fig. 6c, d). To validate the results, siRNA-mediated knockdown of LDH-A was performed in HSCs. The obtained data exhibited that transfection with LDH-A siRNA, similar to oroxylin A treatment alone, apparently disrupted the microfilament skeleton evidenced by cytoskeleton fluorescence staining (Fig. 6e) and reduced MLC2 phosphorylation demonstrated by Western blot assays (Fig. 6f). Additionally, overexpression of LDH-A promoted dense arrangement of cytoskeleton and increased MLC2 phosphorylation, and considerably abrogated oroxylin A-inhibited HSC contraction (Additional file 6: Figure S5). Collectively, these discoveries indicated that inhibition of LDH-A was required for oroxylin A reduction of HSC contraction.

### Oroxylin a alleviates liver fibrotic injury and inhibits HSC glycolysis and contraction by targeting LDH-A in mice intoxicated with CCl_4_

**Fig. 7:**
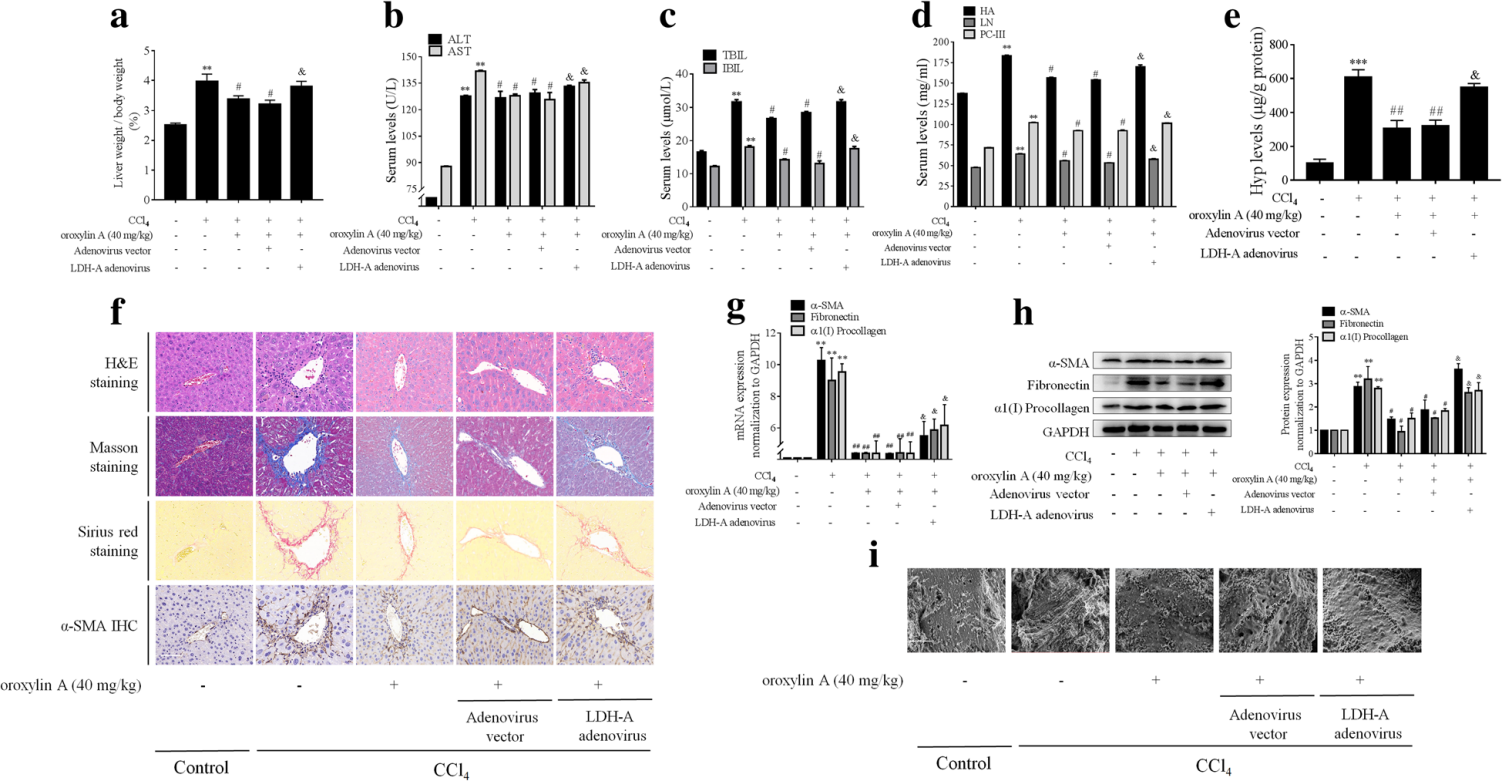
Oroxylin A alleviates liver injury and fibrosis by targeting LDH-A in mice intoxicated with CCl_4_. Animal grouping and treatments refer to the Methods section. **a** Measurements of liver/body weight ratio. **b** Measurements of serum levels of ALT and AST. **c** Measurements of serum levels of TBIL and IBIL. **d** Measurements of serum levels of HA, LN and PC-III. **e** Measurements of hepatic Hyp contents. **f** H&E staining, Masson staining, Sirius red staining, and immunohistochemical analysis of α-SMA in liver tissues, scale bar: 100 μm. **g** Real-time PCR analyses of mRNA expression of α-SMA, fibronectin and α1(I) procollagen in liver tissues. **h** Western blot analyses of protein expression of α-SMA, fibronectin and α1(I) procollagen in liver tissues with quantification. **i** SEM analyses of sinusoidal fenestration indicated by white arrows, scale bar: 2 μm. For statistical significance of this figure: **p < 0.01 vs. control, ***p < 0.001 vs. control; ^#^*p* < 0.05 vs. CCl_4_, ^##^*p* < 0.01 vs. CCl_4_; ^&^*p* < 0.05 vs. CCl_4_ + oroxylin A + adenovirus vector

We used the classical liver fibrosis model induced by intraperitoneal injection of CCl_4_ in mice to establish the in vivo relevance of the above culture-system findings. Because our recent studies have clearly demonstrated that oroxylin A had potent in vivo antifibrotic effects [17, 27], we here focused on testifying whether the effects of oroxylin A were dependent on regulation of LDH-A using adenovirus-mediated overexpression of LDH-A in mice. Oroxylin A reduced the liver/body weight ratio and downregulated the serum levels of hepatocyte injury markers (ALT, AST, TBIL, and IBIL) in fibrotic mice, but these effects of oroxylin A were abolished by overexpression of LDH-A (Fig. 7a-c). Similar changes were observed in the measurements of serum levels of fibrotic markers (HA, LN, and PC-III) and hepatic Hyp contents (Fig. 7d, e). Histological assessments and collagen staining assays showed that oroxylin A amelioration of hepatic structure and collagen deposition was abolished by overexpression of LDH-A in vivo (Fig. 7f). We then examined HSC activation markers and found that oroxylin A significantly reduced the expression of α-SMA, fibronectin and α1(I) procollagen at both mRNA and protein levels in mouse fibrotic liver, but these effects were counteracted by overexpression of LDH-A in fibrotic mice (Fig. 7f-h). Interestingly, SEM data exhibited that treatment with oroxylin A inhibited sinusoidal capillarization and restored the fenestrae of liver sinusoidal endothelial cells in fibrotic mice, but overexpression of LDH-A diminished oroxylin A improvement of hepatic vascular architecture during liver fibrogenesis (Fig. 7i). Altogether, these observations indicated that oroxylin A alleviated liver fibrotic injury by targeting LDH-A in mice.

**Fig. 8:**
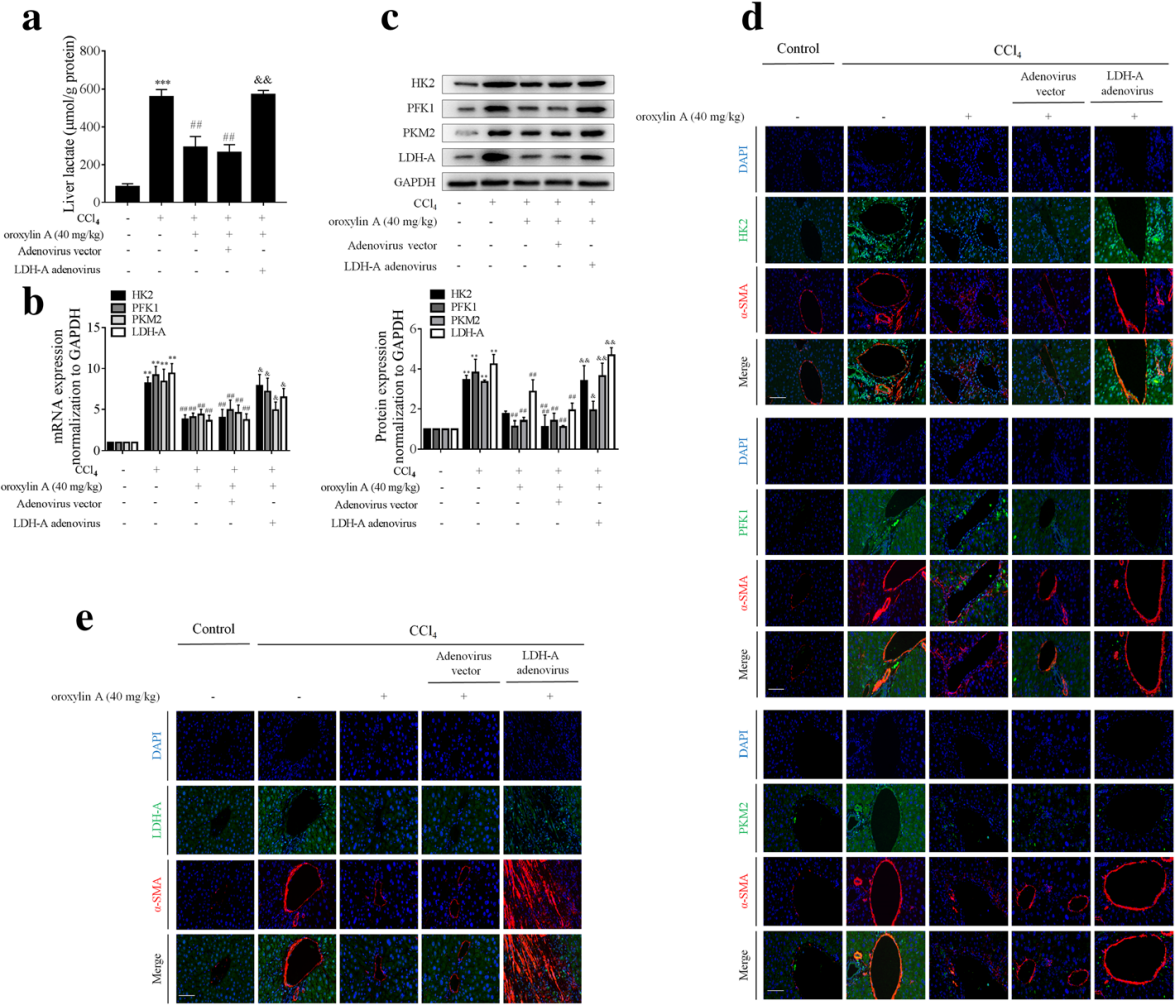
Oroxylin A inhibits HSC glycolysis by targeting LDH-A in mice intoxicated with CCl_4_. Animal grouping and treatments refer to the Methods section. **a** Measurements of hepatic lactate levels. **b** Real-time PCR analyses of mRNA expression of HK2, PFK1, PKM2 and LDH-A in liver tissues. **c** Western blot analyses of protein expression of HK2, PFK1, PKM2 and LDH-A in liver tissues with quantification. **d**, **e** Immunofluorescence analyses of HK2, PFK1, PKM2, and LDH-A in liver tissues. Staining with α-SMA was used to indicate HSCs, scale bar: 20 μm. For statistical significance of this figure: **p < 0.01 vs. control, ***p < 0.001 vs. control; ^##^*p* < 0.01 vs. CCl_4_; ^&^*p* < 0.05 vs. CCl_4_ + oroxylin A + adenovirus vector, ^&&^*p* < 0.01 vs. CCl_4_ + oroxylin A + adenovirus vector

**Fig. 9:**
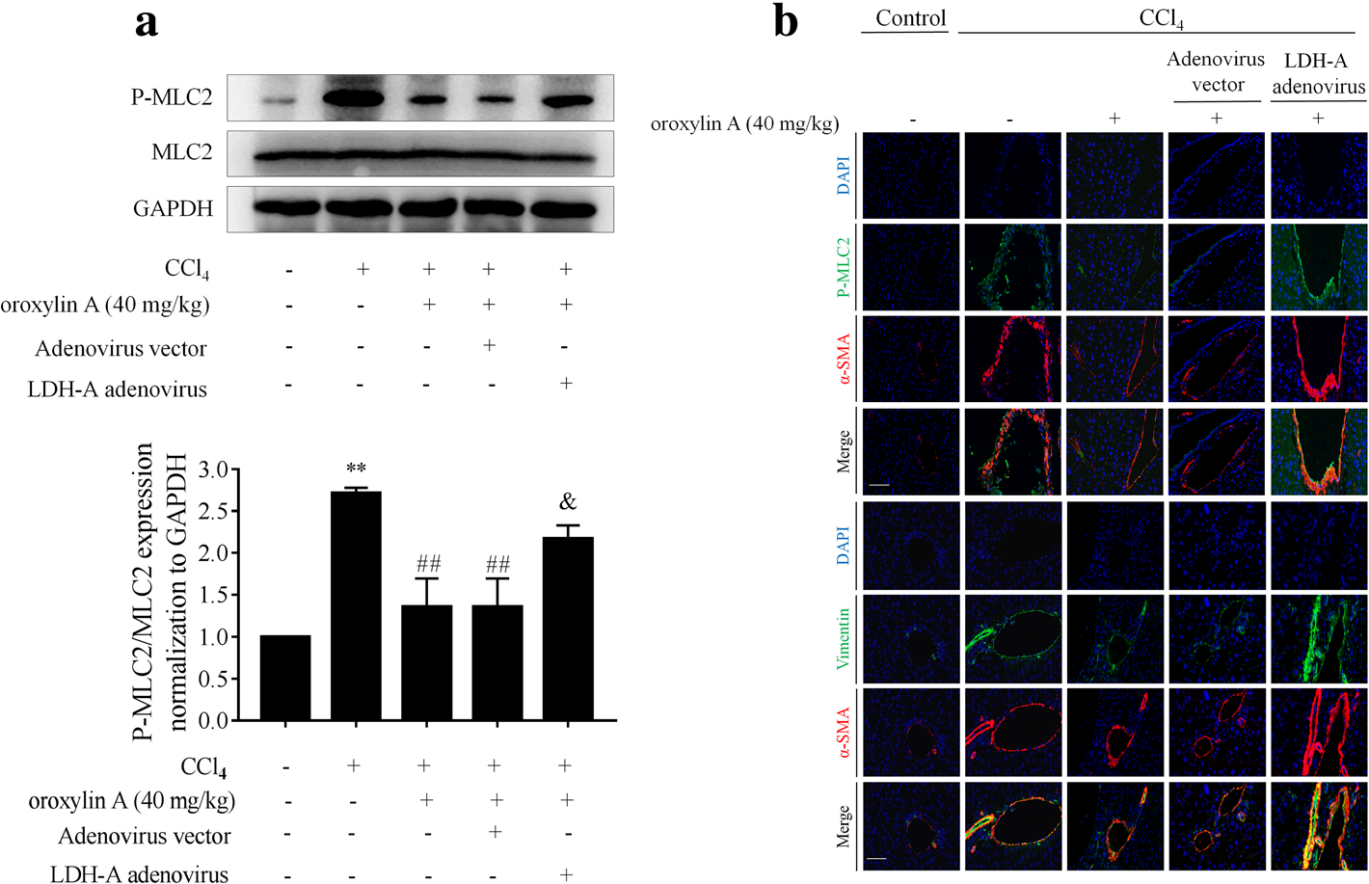
Oroxylin A inhibits HSC contraction by targeting LDH-A in mice intoxicated with CCl_4_. Animal grouping and treatments refer to the Methods section. **a** Western blot analyses of MLC2 phosphorylation in liver tissues with quantification. **b** Immunofluorescence analyses of p-MLC2 and vimentin in liver tissues. Staining with α-SMA was used to indicate HSCs, scale bar: 20 μm. For statistical significance of this figure: **p < 0.01 vs. control; ^##^*p* < 0.01 vs. CCl_4_; ^&^*p* < 0.05 vs. CCl_4_ + oroxylin A + adenovirus vector

We subsequently evaluated the effects of oroxylin A on HSC glycolysis and contraction in fibrotic mice. We observed that hepatic lactate levels in fibrosis model mice were significantly elevated compared to that of control mice, and that oroxylin A intervention considerably decreased hepatic lactate levels, which was abrogated by overexpression of LDH-A (Fig. 8a). Treatment with oroxylin A downregulated the mRNA and protein expression of HK2, PFK1, PKM2 and LDH-A in mouse fibrotic liver, but their reduction was remarkably rescued by overexpression of LDH-A (Fig. 8b, c). Further immunofluorescence analyses with α-SMA staining for indicating HSCs revealed that these key glycolysis rate-limiting enzymes had lower abundance in the HSCs of oroxylin A-treated fibrotic mice compared to the model group, but overexpression of LDH-A impaired the effects of oroxylin A (Fig. 8d, e). We finally examined HSC contraction, and found that MLC2 phosphorylation was significantly increased in mouse fibrotic liver but was decreased by oroxylin A treatment; whereas overexpression of LDH-A rescued oroxylin A-inhibited MLC2 phosphorylation (Fig. 9a, b). Vimentin is a major component of cytoskeleton responsible for stabilization of cytoskeletal interactions [28], and is frequently used as a marker of cell contraction [29]. Here, immunofluorescence analysis of vimentin showed that HSC contraction was enhanced in mouse fibrotic liver but was inhibited by oroxylin A treatment; however, overexpression of LDH-A restored HSC contractile capacity in oroxylin A-treated fibrotic mice (Fig. 9b). Taken together, suppression of HSC glycolysis and contraction by oroxylin A contributed to the reduction of liver fibrosis in mice, and these effects were dependent on inhibition of LDH-A.

## Discussion

HSCs are located in the space of Disse and contact closely with sinusoidal endothelial cells. The contractile phenotype of HSCs has been critically implicated in liver’s response to various injuries, and the density and coverage of HSCs in the sinusoidal lumen are found to be increased during hepatic fibrosis [5]. It is recognized that the enhanced contractility of HSCs increases the resistance in sinusoidal blood flow and aggravates hepatic sinusoidal capillarization and remolding, leading to the development of portal hypertension, a highly lethal complication of advanced chronic liver disease [30]. Accordingly, restriction of HSC contraction represents a novel intervention strategy for liver fibrosis or cirrhosis, as well as portal hypertension. We recently reported that oroxylin A had significant antifibrotic and hepatoprotective effects in vitro and in vivo [17, 31, 32], and observed that oroxylin A could improve sinusoidal vascular remodeling [27]. These observations directed us to investigate whether modulation of HSC contractile phenotype was involved in oroxylin A’s effects. Interestingly, our current data uncovered the association and identified regulation of aerobic glycolysis as a linking molecular event in oroxylin A’ effects.

Accumulating evidence suggests that metabolic reprogramming controls the fate and transdifferentiation of HSCs, and is a conserved response to liver injury. Induction of aerobic glycolysis, similar to the Warburg effect described in tumor cells, has been proven to be a driving force of the dramatic phenotypic alterations of HSCs during hepatic repair, including the high proliferative and fibrogenic activities [12]. This phenomenon can be explained by the fact that glycolysis produces ATP at a faster rate than oxidative phosphorylation, although it only generates two ATP molecules per molecule of glucose. Glycolysis thus is a faster and shorter pathway for energy generation used by some cells to meet the high demands of rapid proliferation [33]. This metabolic switch has important therapeutic relevance and implication for liver fibrosis. Indeed, our previous work demonstrated that the well-known natural product curcumin inhibited HSC activation and reduced hepatic fibrosis through disrupting aerobic glycolysis [34, 35]. In current work, we postulated that the contractile phenotype of HSCs could also be governed by aerobic glycolysis and drug-induced metabolic perturbation could affect HSC contraction and related pathology in liver fibrosis. We found that oroxylin A potently inhibited HSC contraction evidenced by interruption of cytoskeleton arrangement and reduced MLC2 phosphorylation, and meanwhile, the glycolytic flux and activity were effectively blocked by oroxylin A evidenced by reduced glucose uptake and consumption, decreased lactate production and downregulation of three key rate-limiting enzymes. More importantly, we identified that oroxylin A blockade of aerobic glycolysis contributed to the restriction of HSC contraction. This point was easily understandable, because many components of the contraction machinery are involved in the efficient coupling of energy source and dependent on myosin-actin interaction using ATP [36]. The energy-contraction coupling was disrupted by blockade of aerobic glycolysis and reduction of energy supply in oroxylin-treated HSCs.

We subsequently investigated the potential upstream molecule mediating oroxylin A disruption of the energy-contraction coupling machinery. We focused on the role of LDH-A because of the following points. (i) LDH-A was highly expressed in human fibrotic liver, implying a close association between LDH-A and hepatic fibrogenesis. (ii) LDH-A converts pyruvate, the final product of glycolysis, to lactate, shifting the use of glucose metabolites from simple energy production to acceleration of cell growth and replication, and thus LDH-A activity has been characterized as a promising target in cancer therapy by preventing cancer cells from proliferating [10]. (iii) LDH-A was newly recognized as a regulator of gene transcription via translocating into nucleus and binding to DNA, and phosphorylation of LDH at Tyr238 has been characterized to be important for its nuclear translocation [37]. Here, we observed that oroxylin A suppressed the expression and activity of LDH-A in HSCs, and, using chemical and genetic approaches, confirmed that inhibition of LDH-A was a prerequisite for oroxylin A reduction of glycolysis-dependent HSC contraction and liver fibrosis in vitro and in vivo. These results raised an interesting question that why modulation of LDH-A could be the causative event in this context given that LDH-A works at the final stage of glycolysis pathway. We postulated that this could be explained by two reasons. (i) Inhibition of LDH-A by oroxylin A synergistically blocked the glycolytic flux, leading to the reduced energy production and resultant restriction of contraction. (ii) LDH-A could regulate the expression of glycolysis rate-limiting enzymes such as HK2, PFK1 and PKM2. LDH-A might act as a transcription factor or co-activator to increase the transcription of these enzymes. This speculation could be, at least partially, supported by the observation that the de novo synthesis of these enzymes was inhibited by oroxylin A, blocking each rate-limiting step of glycolysis. We understand that our results could not rule out the possibility that the expression of these enzymes was inhibited by oroxylin A directly, or indirectly by targeting other molecules, given the fact that natural products commonly have multiple targets within cells.

## Conclusions

In conclusion, our current work linked the aerobic glycolysis pathway to the contractile phenotype of HSCs, and uncovered that oroxylin A blocked glycolysis-dependent HSC contraction and reduced hepatic fibrosis through inhibition of LDH-A. We suggested LDH-A as a promising target for disruption of HSC metabolism implicated in liver fibrosis therapy.

## Additional files

Additional file 1: **Table S1.** Primer sequences for real-time PCR. (DOCX 16 kb)

Additional file 2: **Figure S1.** Oroxylin A reduces the intracellular ATP levels in HSCs. LX2 cells were treated with oroxylin A at indicated concentrations for 24 h. Measurements of intracellular ATP levels. For statistical significance of this figure: *p < 0.05 vs. control. (TIF 43 kb)

Additional file 3: **Figure S2.** Effects of 2-DG or galloflavin cell viability using MTT assays. a LX2 cells were treated with 2-DG at indicated concentrations for 24 h. b Hepatocyte LO2 cells were treated with 2-DG at indicated concentrations for 24 h. c LX2 cells were treated with galloflavin at indicated concentrations for 24 h. b Hepatocyte LO2 cells were treated with galloflavin at indicated concentrations for 24 h. For statistical significance of this figure: *p < 0.05 vs. control, **p < 0.01 vs. control. (TIF 68 kb)

Additional file 4: **Figure S3.** Real-time PCR analyses of mRNA expression of LDH-A in human healthy liver or fibrotic liver. For statistical significance of this figure: *p < 0.05 vs. healthy liver .(TIF 43 kb)

Additional file 5: **Figure S4.** LX2 cells were transfected with LDH-A overexpression plasmid and/or treated with oroxylin A at indicated concentrations for 24 h. a Western blot analyses of protein expression of LDH-A. b Western blot analyses of protein expression of HK2, PFK1 and PKM2. (TIF 104 kb)

Additional file 6: **Figure S5.** LX2 cells were transfected with LDH-A overexpression plasmid and/or treated with oroxylin A at indicated concentrations for 24 h. a Western blot analyses of MLC2 phosphorylation. b Cytoskeleton fluorescence staining, scale bar: 20 μm (TIF 793 kb)

## Abbreviations

2-DG: 2-Deoxy-D-glucose
ALT: Alanine aminotransferase
AST: Aspartate aminotransferase
CCl_4_: Carbon tetrachloride
DAPI: Diamidino-phenyl-indole
ECAR: Extracellular acidification rate
GAPDH: Gglyceraldehyde phosphate dehydrogenase
GOD: Glucose oxidase
H&E: Hematoxylin-eosin
HA: Hyaluronic acid
HK2: Hexokinase 2
HSC: Hepatic stellate cell
Hyp: Hydroxyproline
IBIL: Indirect bilirubin
LDH: Lactate dehydrogenase
LN: Laminin
MLC2: Myosin light chain 2
PC-III: Procollagen type III
PFK1: Phosphofructokinase 1
PKM2: Pyruvate kinas type M2
SEM: Scanning electronic microscopy
TBIL: Total bilirubin
α-SMA: α-Smooth muscle actin
