# Supplementary material for: Blockade of glycolysis-dependent contraction by oroxylin a via inhibition of lactate dehydrogenase-a in hepatic stellate cells
Source: Cell Commun Signal. 2019 Feb 11;17:11. doi: 10.1186/s12964-019-0324-8 (PMC6371416; doi:10.1186/s12964-019-0324-8)
Supplement: Supplementary file 1 — Table S1. Primer sequences for real-time PCR. (DOCX 16 kb) [file 12964_2019_324_MOESM1_ESM.docx]

**Table S1** Primer sequences for Real-time PCR

| Gene | Forward sequence | Reverse sequence |
| --- | --- | --- |
| Human-HK2 | 5’-GAGCCACCACTCACCCTACT-3’ | 5’-ACCCAAAGCACACGGAAGTT-3’ |
| Human-PFK1 | 5’-GGGGATGCTCAAGGTATGAAC-3’ | 5’-TCGGCCTCTGCGATGTTTG-3’ |
| Human-PKM2 | 5’-TACCATGCGGAGACCATCAA-3’ | 5’-AGCAACGGGCCGGTAGAG-3’ |
| Human-LDHA | 5’-TTGGTCCAGCGTAACGTGAAC-3’ | 5’-CCAGGATGTGTAGCCTTTGAG-3’ |
| Human-GAPDH | 5’-CCAACCGCGAGAAGATGA-3’ | 5’-CCAGAGGCGTACAGGGATAG-3’ |
| Mouse-HK2 | 5’-GGGTAGCCACGGAGTACAAA-3’ | 5’-TGGATTGAAAGCCAACTTCC-3’ |
| Mouse-PFK1 | 5’-GCCGTGAAACTCCGAGGAA-3’ | 5’-GTTGCTCTTGACAATCTTCTCATCAG-3’ |
| Mouse-PKM2 | 5’-TGGGATGGAAACTGTGAAGAG-3’ | 5’-CGGAGTTCCTCGAATAGCTG-3’ |
| Mouse-LDH-A | 5’-ATGCACCCGCCTAAGGTTCTT-3’ | 5’-TGCCTACGAGGTGATCAAGCT-3’ |
| Mouse-α-SMA | 5’-AAGAGCATCCGACACTGCTGAC-3’ | 5’-AGCACAGCCTGAATAGCCACATAC-3’ |
| Mouse-Fibronectin | 5’-GCTTTGGCAGTGGTCATTTCAG-3’ | 5’-ATTCCCGAGGCATGTGCAG-3’ |
| Mouse-α1(I)Procollagen | 5’-CCTGGACGCCATCAAGGTCTAC-3’ | 5’-CCAAGTTCCGGTGTGACTCG-3’ |
| Mouse-GAPDH | 5’-GTCTGGAGAAACAGCCAAGG-3’ | 5’-AGAGCACCAGAGGAGGACG-3’ |
