# Supplementary figures and images for: Blockade of glycolysis-dependent contraction by oroxylin a via inhibition of lactate dehydrogenase-a in hepatic stellate cells
Source: Cell Commun Signal. 2019 Feb 11;17:11. doi: 10.1186/s12964-019-0324-8 (PMC6371416; doi:10.1186/s12964-019-0324-8)

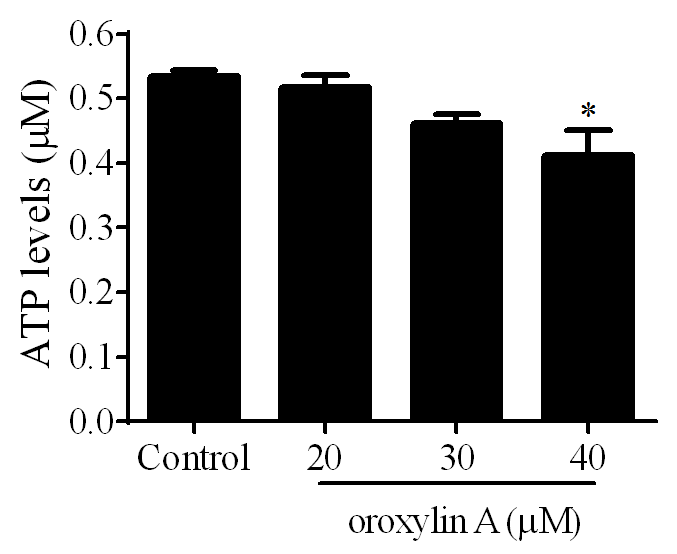

Supplement: Supplementary file 2 — Figure S1. Oroxylin A reduces the intracellular ATP levels in HSCs. LX2 cells were treated with oroxylin A at indicated concentrations for 24 h. Measurements of intracellular ATP levels. For statistical significance of this figure: *p < 0.05 vs. control. (TIF 43 kb) [file 12964_2019_324_MOESM2_ESM.tif]

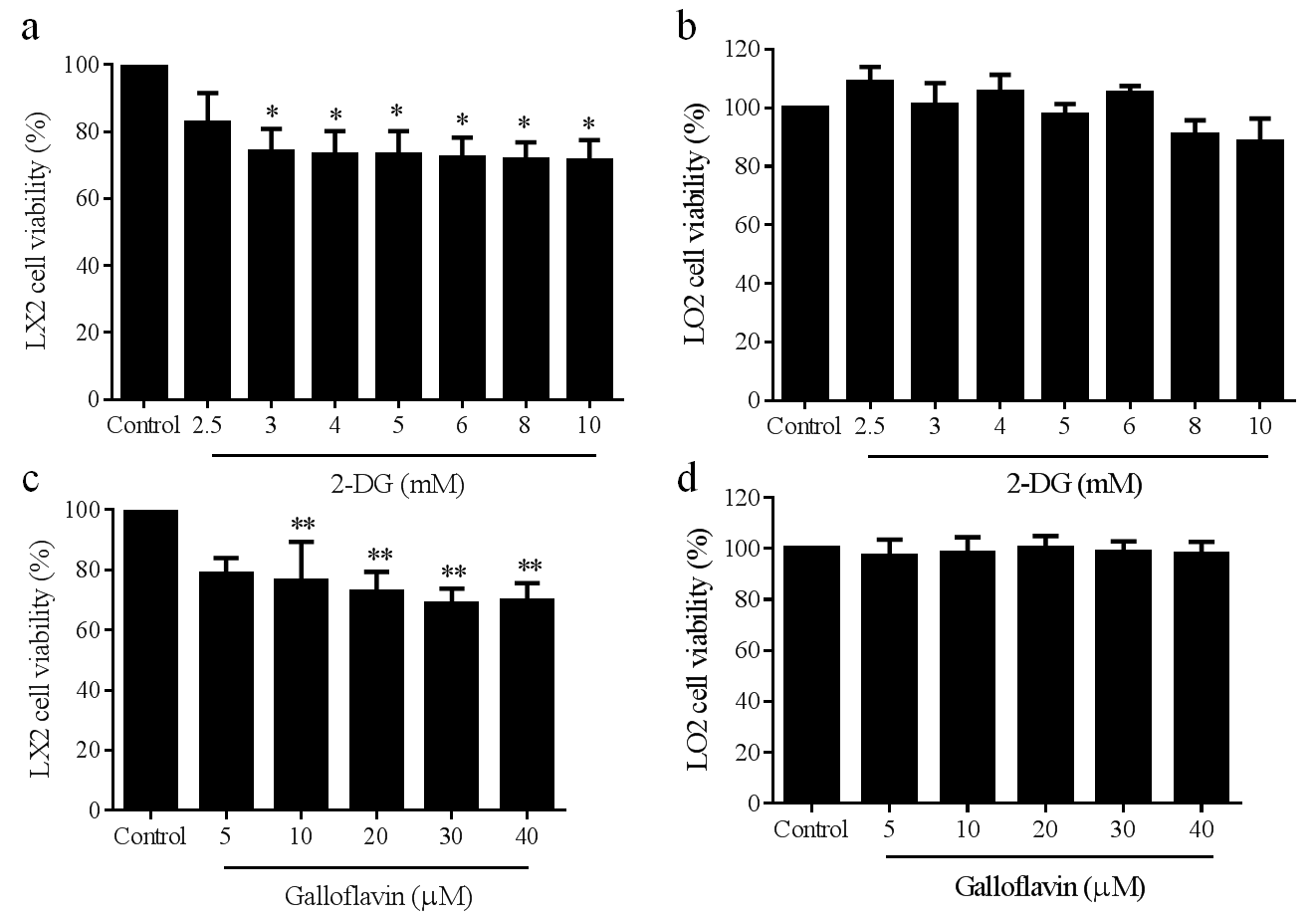

Supplement: Supplementary file 3 — Figure S2. Effects of 2-DG or galloflavin cell viability using MTT assays. a LX2 cells were treated with 2-DG at indicated concentrations for 24 h. b Hepatocyte LO2 cells were treated with 2-DG at indicated concentrations for 24 h. c LX2 cells were treated with galloflavin at indicated concentrations for 24 h. b Hepatocyte LO2 cells were treated with galloflavin at indicated concentrations for 24 h. For statistical significance of this figure: *p < 0.05 vs. control, **p < 0.01 vs. control. (TIF 68 kb) [file 12964_2019_324_MOESM3_ESM.tif]

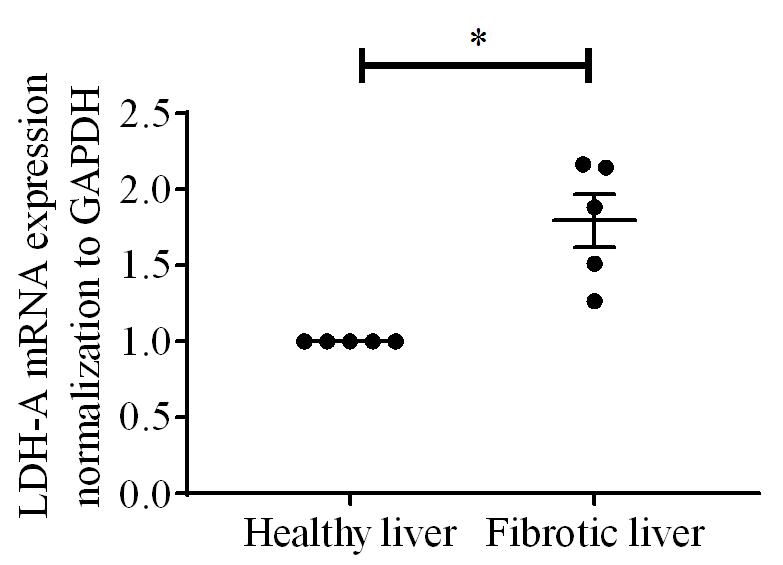

Supplement: Supplementary file 4 — Figure S3. Real-time PCR analyses of mRNA expression of LDH-A in human healthy liver or fibrotic liver. For statistical significance of this figure: *p < 0.05 vs. healthy liver .(TIF 43 kb) [file 12964_2019_324_MOESM4_ESM.tif]

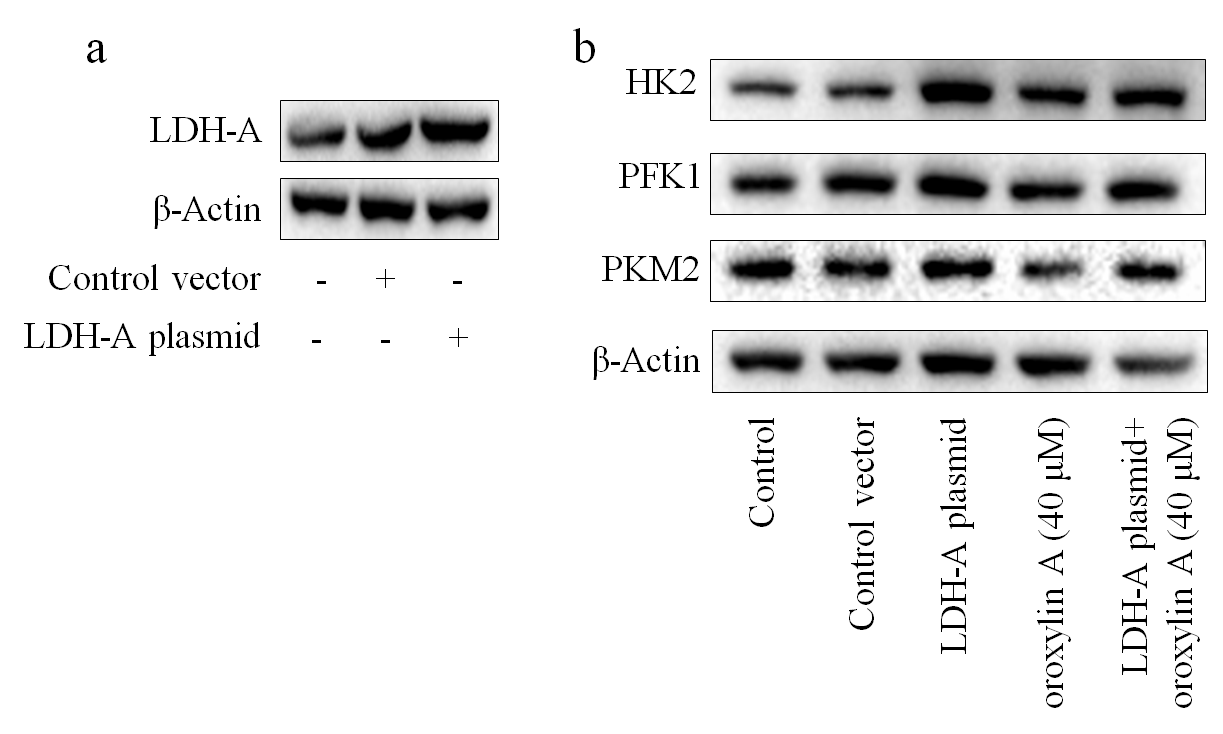

Supplement: Supplementary file 5 — Figure S4. LX2 cells were transfected with LDH-A overexpression plasmid and/or treated with oroxylin A at indicated concentrations for 24 h. a Western blot analyses of protein expression of LDH-A. b Western blot analyses of protein expression of HK2, PFK1 and PKM2. (TIF 104 kb) [file 12964_2019_324_MOESM5_ESM.tif]

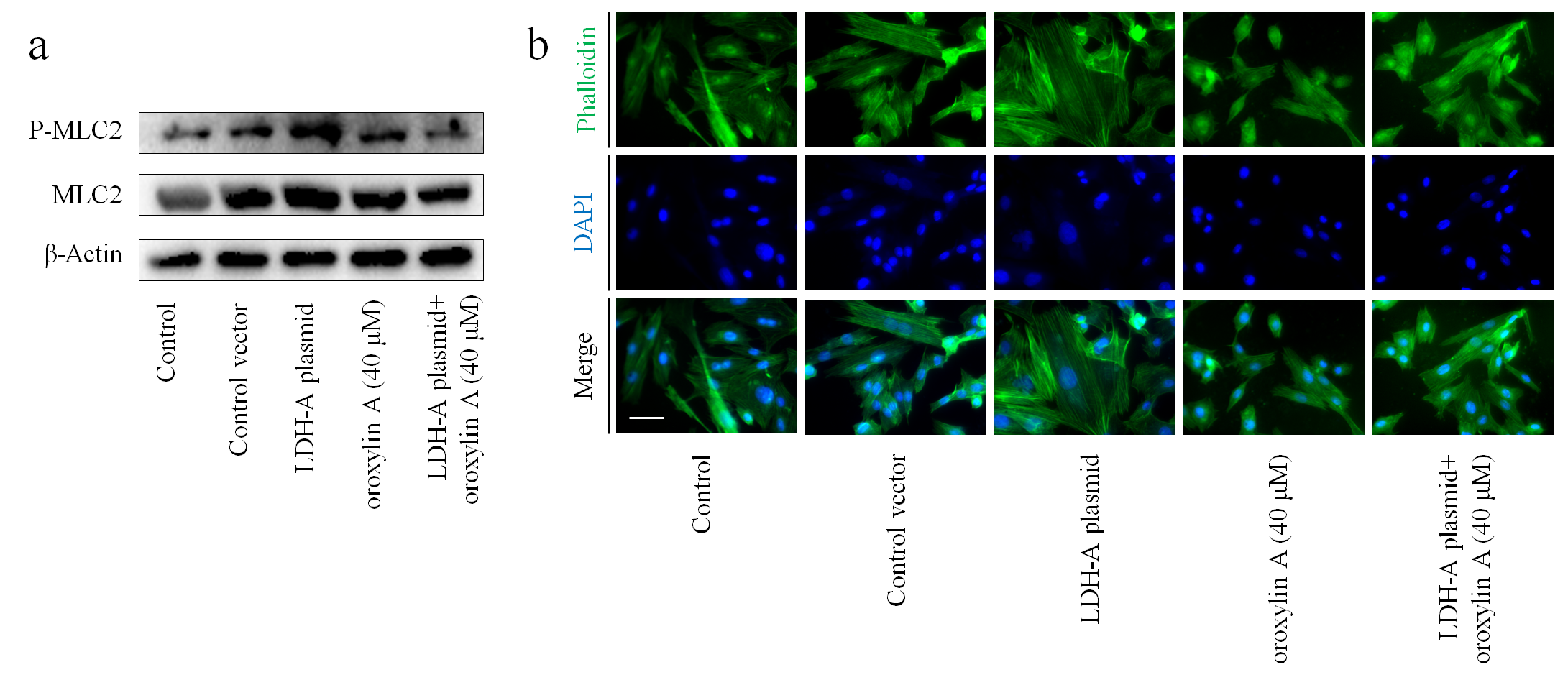

Supplement: Supplementary file 6 — Figure S5. LX2 cells were transfected with LDH-A overexpression plasmid and/or treated with oroxylin A at indicated concentrations for 24 h. a Western blot analyses of MLC2 phosphorylation. b Cytoskeleton fluorescence staining, scale bar: 20 μm (TIF 793 kb) [file 12964_2019_324_MOESM6_ESM.tif]
